# DNA Methylation Cancer Biomarkers: Translation to the Clinic

**DOI:** 10.3389/fgene.2019.01150

**Published:** 2019-11-14

**Authors:** Warwick J. Locke, Dominic Guanzon, Chenkai Ma, Yi Jin Liew, Konsta R. Duesing, Kim Y.C. Fung, Jason P. Ross

**Affiliations:** ^1^Molecular Diagnostics Solutions, CSIRO Health and Biosecurity, North Ryde, NSW, Australia; ^2^Probing Biosystems Future Science Platform, CSIRO Health and Biosecurity, Canberra, ACT, Australia

**Keywords:** DNA methylation, diagnostic, translation, cancer, epigenetics, liquid biopsy

## Abstract

Carcinogenesis is accompanied by widespread DNA methylation changes within the cell. These changes are characterized by a globally hypomethylated genome with focal hypermethylation of numerous 5’-cytosine-phosphate-guanine-3’ (CpG) islands, often spanning gene promoters and first exons. Many of these epigenetic changes occur early in tumorigenesis and are highly pervasive across a tumor type. This allows DNA methylation cancer biomarkers to be suitable for early detection and also to have utility across a range of areas relevant to cancer detection and treatment. Such tests are also simple in construction, as only one or a few loci need to be targeted for good test coverage. These properties make cancer-associated DNA methylation changes very attractive for development of cancer biomarker tests with substantive clinical utility. Across the patient journey from initial detection, to treatment and then monitoring, there are several points where DNA methylation assays can inform clinical practice. Assays on surgically removed tumor tissue are useful to determine indicators of treatment resistance, prognostication of outcome, or to molecularly characterize, classify, and determine the tissue of origin of a tumor. Cancer-associated DNA methylation changes can also be detected with accuracy in the cell-free DNA present in blood, stool, urine, and other biosamples. Such tests hold great promise for the development of simple, economical, and highly specific cancer detection tests suitable for population-wide screening, with several successfully translated examples already. The ability of circulating tumor DNA liquid biopsy assays to monitor cancer *in situ* also allows for the ability to monitor response to therapy, to detect minimal residual disease and as an early biomarker for cancer recurrence. This review will summarize existing DNA methylation cancer biomarkers used in clinical practice across the application domains above, discuss what makes a suitable DNA methylation cancer biomarker, and identify barriers to translation. We discuss technical factors such as the analytical performance and product-market fit, factors that contribute to successful downstream investment, including geography, and how this impacts intellectual property, regulatory hurdles, and the future of the marketplace and healthcare system.

## Introduction

Cancer is defined by extensive genetic changes and associated dysregulation in gene function and activity (Nakagawa and Fujita, 2018). However, cancer is not an exclusively genetic disease and its progression is dependent on a host of additional biological processes such as immune activity, the tissue microenvironment, and epigenetics (Hanahan and Weinberg, 2011). Epigenetics is a second layer of information encoded onto the genome that guides genomic function and activity. Epigenetics acts through two mechanisms: (1) modifications to chromosomal proteins that alter the 3D conformation of the genome and/or protein-DNA interactions and (2) chemical modification of the DNA strand itself (Kondo, 2009). Change in the 3D structure of DNA is enacted *via* post-translational modifications of the histone proteins at the center of the simplest DNA structure, the nucleosome. Histone modifications can lead to either tightly packed and inactive conformations or open and accessible DNA (termed heterochromatin and euchromatin respectively). The best characterized chemical modification of DNA is the methylation of cytosine to 5-methylcytosine (5mC) that occurs almost exclusively in the context of a cytosine base linked by the DNA phosphate-backbone to guanosine, termed a CpG site. DNA methylation is considered a “soft” and potentially reversible change to the genome that can define or adapt to tumor biology and is functionally equivalent to genetic changes like mutation or deletion (Kulis and Esteller, 2010).

Epigenetic changes are considered to be among the earliest and most comprehensive genomic aberrations occurring during carcinogenesis (Alvarez et al., 2011) and reviewed in (Feinberg et al., 2006). These changes can be broadly characterized as focal hypermethylation and global hypomethylation (Ross et al., 2010). Each mechanism has their own role to play in defining carcinogenesis. Hypomethylation occurs predominantly at repetitive regions and has been demonstrated to be a carcinogenic process in its own right (Gaudet et al., 2003). Hypomethylation also promotes genomic instability, causing missegregation of chromosomes during cell division (Prada et al., 2012) and the unwanted activation of transposable elements within the genome, leading to further genetic damage (Daskalos et al., 2009). Hypermethylation can drive the silencing of key tumor suppressors (Belinsky et al., 1998) or regulatory regions within the genome leading to dysregulation of cell growth or altered response to cancer therapies (Stone et al., 2015). Such epigenetic mechanisms can synergize with known driver mutations to facilitate cancer development or evolution (Tao et al., 2019). Despite the varied and complex nature of changes to the epigenetic landscape, many cancers exhibit a high degree of concordance across tissues, or within the tissue of origin (Zhang and Huang, 2017; Yang et al., 2017b; Hoadley et al., 2018). The robust and common nature of DNA methylation aberrations in cancer and the stability of cell-free DNA in body fluids are attractive properties for diagnostic development. The widespread nature of epigenetic change across the genome can also facilitate increases in sensitivity and specificity by utilizing multiple target loci in a single assay. When combined with the informative nature of these changes regarding cancer biology, DNA methylation-based biomarkers have great potential to transform the treatment and observation of cancer and other diseases.

**Figure 1:**
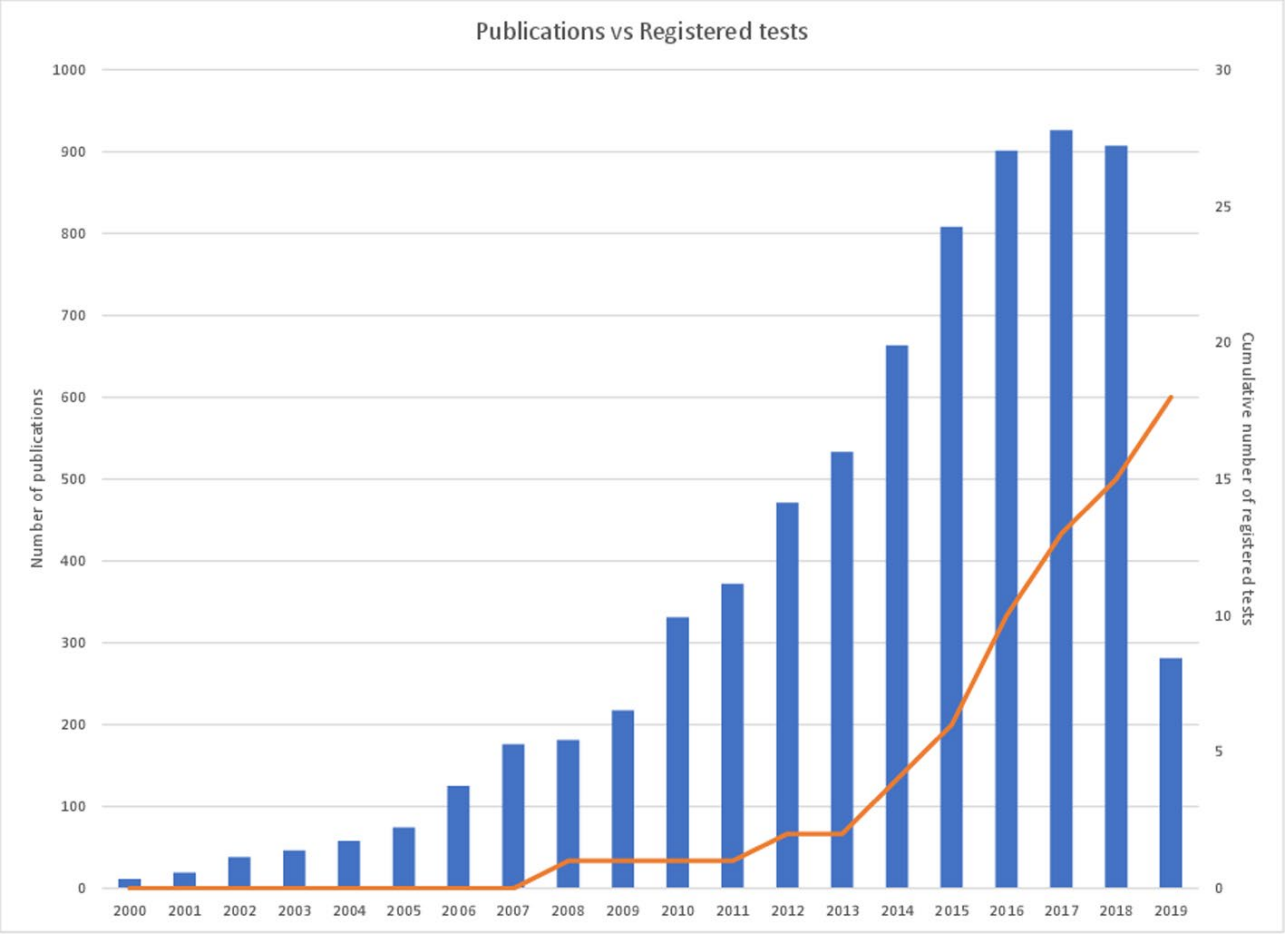
Cancer epigenetic biomarker publications per annum versus cumulative registered DNA-methylated based IVDs. The figure demonstrates the number of cancer epigenetic biomarker academic publications per annum over the last 20 years (left axis) in comparison with the cumulative number of registered cancer epigenetic diagnostic tests available on the market (right axis). A PubMed search utilizing the term ‘epigenetic biomarkers cancer’ was used to determine the number of publications per year and the number of registered tests is referenced in **Table 1**.

The value of epigenetic changes as candidate biomarkers is reflected in the scientific literature with thousands of studies published to date that associate DNA methylation with clinical parameters. However, there is a paucity of markers that have been successfully translated into clinical practice (**Figure 1**). Historically, this has in part been due to limitations of technology to assess epigenetic information at a large scale or in a cost-effective manner. Recent improvements in DNA sequencing and other molecular technologies have helped overcome these initial barriers. However, translation is still a slow and costly process. In this review, we will discuss the current state of the DNA methylation biomarker landscape, the current barriers to translation (be they scientific or regulatory), and what the future may look like for this emerging field of diagnostics.

## Designing an Effective Assay

### Clinical Utility

Traditional diagnostic approaches based on clinical pathology utilize patient biopsied cancerous tissue. Histological analysis of tumor specimens has long been the gold standard for tumor subtyping and diagnosis. Modern epigenetic methods may also make use of such samples, allowing for novel molecular diagnostics to be run in parallel to traditional techniques. DNA methylation analysis does not require any special handling of tumor specimens and can also be applied with similar efficiency to fresh frozen and formalin fixed paraffin embedded tissue. Indeed, early market offerings in the DNA methylation oncology diagnostic space were based upon detecting hypermethylated DNA using fresh tumor biopsies or fixed tissue blocks in glioblastoma, prostate, and colorectal cancer (CRC) (e.g. *MGMT*, *GSTP1*, and *MLH1* based assays) (Esteller et al., 1998; Herman et al., 1998; Esteller et al., 2000).

DNA methylation analysis is not limited to tissue specimens and can be readily extended to almost any bodily fluid (typically termed a “liquid biopsy”). Various bodily fluids contain a host of informative molecules linked to tumorigenesis, growth, immune/cancer interactions, and cell death, circulating tumor cells (CTC) and microvesicles such as exosomes (Wang et al., 2017). These molecules are easily assayed using non- or minimally-invasive techniques and are of extremely high value where tumor tissue, from surgery or biopsy, is not available. Circulating tumor DNA (ctDNA), which is the cancer-originating component of cell free DNA (cfDNA), can provide a window into a tumors mutational and epigenetic profile and has a range of benefits over a traditional tissue biopsy approach (Gai and Sun, 2019).

Simple tissue biopsies only sample a subpopulation of all cell types and with intra-tumoral heterogeneity/clonality could provide a misleading image of the true cellular makeup of the tumor. Recent studies indicate that ctDNA may better capture this natural variation by facilitating sampling of a broader proportion of tumor cells (Dagogo-Jack and Shaw, 2018). This is due to unbiased nature of ctDNA, in that all cell types are likely to make some contribution to the total DNA population. While many registered translated tests [i.e. those with Food and Drug Administration (FDA) pre-market approval (PMA), a European CE mark (CE-IVD) or registered as a lab-developed test (LDT) through the Centers for Medicare and Medicaid Services [CMS]] utilize ctDNA as their target (**Table 1**), ctDNA is not a cure-all for current diagnostic shortfalls. Circulating DNA from rare tumor sub-populations may only be present in ctDNA at vanishingly small levels, making it difficult to detect by even the most sensitive methods. Such limitations should be considered when designing new assays or assessing diagnostic results from liquid biopsy. Despite this, with the right biomarker it is possible to design a simple liquid biopsy that can detect cancer or tumor characteristics with excellent sensitivity. Additionally, such an assay may be run serially with minimal impact on patients, even where biopsy is impossible or impractical, such as during advanced metastatic disease. Overall, from an efficacy and translation standpoint, liquid biopsy is an extremely attractive strategy with the capacity to greatly transform disease diagnosis and management in the near future.

**Table 1:** Current registered liquid biopsy tests in the marketplace.

Test name	Tissue	Biomarker(s)	Biosample	Population	Intended clinical use	Manufacturer/Distributor	Approval
AssureMDx™	Bladder	*TWIST1*, *ONECUT2*, *OTX1* (+ *FGFR3*, *TERT*, *HRAS* mutations)	Voided urine	Patients diagnosed with hematuria	Detection of bladder cancer to avoid cystoscopy	MdxHealth	2017*
Bladder CARE™	Bladder	*SOX1*, *IRAK3* and methylated LINE1	Voided urine	Patients with a history of bladder cancer, smokers, and specific occupations, not currently included in a bladder cancer screening program	Detection of bladder cancer	Pangea	2019*
Bladder EpiCheck^®^	Bladder	Score over 15 methylation markers	Voided urine	Monitoring for tumor recurrence in patients previously diagnosed with bladder cancer	Surveillance of non-muscle-invasive bladder cancer (NMIBC)	Nucleix	2017†
therascreen^®^ PITX2 RGQ	Breast	*PITX2*	Formalin fixed paraffin-embedded (FFPE) tumor tissue taken from primary lesions	Lymph node-positive, ER+, HER2− high-risk breast cancer patients treated with anthracycline chemotherapy	Predict response to anthracycline-based chemotherapy	Qiagen	2018†
IvyGene^®^	Breast, colon, liver, lung	Score	Blood, 40 ml	Direct to consumer	Detection of cancer	Laboratory for Advanced Medicine	2018*
GynTect^®^	Cervical	*ASTN1*, *DLX1*, *ITGA4*, *RXFP3*, *SOX17*, *ZNF671*	Cervical smear in STM medium	Women who are HPV-positive with abnormal cytology findings (Pap III, Pap IIID)	Triage of unclear cervical cancer screening tests	Oncgnostics	2019†
QIAsure	Cervical	*FAM19A4*, *hsa-mir124-2*	Cervical scrape, vaginal sample	Women who are high-risk HPV positive or have ASC-US cytology	Triage of unclear cervical cancer screening tests	Qiagen	2016†
Cologuard^®^	Colorectal	*NDRG4*, *BMP3* (+ *KRAS* mutation, occult hemoglobin)	Stool	Patients, 50 years and older, at average risk who are typical candidates for CRC screening	Detection of colorectal cancer (CRC)	Exact Sciences	2014‡
ColoSure™	Colorectal	*VIM*	Stool	Patients unwilling or unable to undergo a more invasive exam	Detection of CRC	LabCorp	2008*
COLVERA™	Colorectal	*IKZF1*, *BCAT1*	Plasma, 3.9 ml	Detect both residual disease and recurrent disease in CRC patients	Detection of residual disease post-surgical resection, for surveillance of recurrent CRC after primary treatment	Clinical Genomics	2016*
Epi proColon^®^	Colorectal	*SEPT9*	Plasma, 3.5 ml	Patients, 50 years or older, with average risk for CRC, who decline other CRC screening	Detection of CRC	Epigenomics	2016†,‡
Human MGMT Gene Methylation Detection	Glioblastoma	*MGMT*	Tumor biopsy	Glioblastoma patients	Predict response to alkylating agent chemotherapy such as Temozolomide	Xiamen SpacegenCo	2016†
PredictMDx™	Glioblastoma	*MGMT*	Tumor biopsy	Glioblastoma patients	Predict response to alkylating agent chemotherapy such as Temozolomide	LabCorp	2012*
therascreen^®^ MGMT Pyro^®^	Glioblastoma	*MGMT*	Blood ctDNA or FFPE Tumor biopsy	Glioblastoma patients	Predict response to alkylating agent chemotherapy such as Temozolomide	Qiagen	2015†
HCCBloodTest	Liver	*SEPT9*	Plasma, 3.5 ml	Patients with cirrhosis	Detection of hepatocellular carcinoma	Epigenomics	2019†
Epi proLung^®^	Lung	*SHOX2*, *PTGER4*	Plasma, 3.5 ml	Increased risk patients defined by life history, presentation with symptoms, radiological findings in the lung	Detection of lung cancer in patients at increased risk for the disease	Epigenomics	2017†
ConfirmMDx	Prostate	*GSTP1*, *APC*, *RASSF1*	Prostate biopsy	Men with established risk factors	Detection of occult prostate cancer on previously biopsied, histopathologically negative tissue	MdxHealth	2012*
EPICUP™	Unknown Origin	Human Methylation450 BeadChip	Fresh frozen or FFPE tumor biopsy	Patients with cancer of unknown primary (CUP) origin	Predict cancer tissue of origin to enable direction of tumor type-specific therapy	Ferrer	2015†

*CLIA LDT, †CE-IVD, ‡FDA PMA

HPV, human papillomavirus; ASC-US, atypical squamous cells of undetermined significance.

There are at least six broad diagnostic areas in which a DNA methylation cancer liquid biopsy test may be combined with traditional screening and medical imaging for better patient outcomes:

1. Primary diagnosis: determine individuals potentially presenting with cancer and who should be followed up by traditional screening exams.
2. Triage: after indeterminate results from imaging or biopsy, a further test to decide invasive and/or non-invasive follow-up.
3. Choice of therapy: diagnostics which influence the choice of treatment. This can include prognostic markers, which grade tumors as likely to be treatable with first-line therapy, or companion diagnostics, in which a test result is linked with efficacy of a particular treatment (may also be based on direct tumor biopsy).
4. Response to therapy and treatment failure: the measurement of ctDNA tumor load in the blood to monitor the initial response to therapy and to detect a later rise in ctDNA load consistent with subsequent resistance to therapy. Testing can be serial to detect trend.
5. Residual disease monitoring: for determining minimal residual disease after surgery, adjuvant chemotherapy or radiotherapy and identify patients at increased risk of disease recurrence.
6. Recurrence: early detection of recurrence to give more opportunity for treatment with curative intent.

Most existing tests for cancer screening, diagnosis, or monitoring are protein immunoassays or imaging. For example, many countries have adopted the prostate specific antigen (PSA) test as a population screen for prostate cancer; and the fecal occult blood test (FOBT) or improved fecal immunochemical test (FIT) for population screening of CRC. Although inexpensive and widely used, none of the screening or recurrence tests have the ideal performance characteristics for their respective cancer type, providing opportunity for development of alternate tests, such as DNA methylation tests, to better inform clinical management.

### Performance Characteristics

The six diagnostic areas above are best addressed with a blend of liquid biopsy technologies. For population screening (primary diagnosis and triage) the diagnostic must be inexpensive, non-invasive, reliable, and have high specificity to reduce false positive results and unnecessary follow-up procedures. With residual disease monitoring and recurrence, the diagnostic test should exhibit high sensitivity. Ideally, response to therapy and treatment failure diagnostic tests should be rapid and inexpensive. The response area is well suited for point-of-care devices which allow immediate decision-making about treatment efficacy and allow inexpensive serial testing to quickly flag the onset of resistance to current therapy. Choice of therapy diagnostics are tailored to fit clinical decision-making around treatment and can also be designed as companion diagnostics developed in conjunction with a partnered therapeutic intervention.

Going forward, the general nature of DNA methylation ctDNA diagnostics and their economic and high-throughput nature suggests these markers will continue to have a growing role in the six broad areas outlined above. While somatic mutation screening surveys of ctDNA using next-generation sequencing (NGS) and the examination of CTCs are more expensive, they offer unique insight around treatment options and the development of resistance as these approaches can reveal “druggable mutations”. The economics of the therapy response market support these expensive tests; often this precision oncology information informs whether the prescription of expensive chemotherapy drugs will be efficacious.

### Combining With Other Modes of Detection

The performance of liquid biopsy ctDNA somatic mutation tests is reduced in earlier stage tumors, likely due to far lower levels of ctDNA in the blood (Bettegowda et al., 2014). Reduced sensitivity for earlier stage tumors is also observed with DNA methylation-based liquid biopsy tests of ctDNA, even though the methylation changes are apparent in early stage tumor sections (Church et al., 2014; Pedersen et al., 2015a). For example, *BCAT1* and *IKZF1* are hypermethylated in 97.8% and 86.8% of CRC tumor biopsies, respectively; yet, the ability to detect ctDNA using the same assay vary by staging, tumor size, location, and lymphatic invasion (Pedersen et al., 2015a; Symonds et al., 2016; Jedi et al., 2018; Symonds et al., 2018). Early stage tumors are not highly vascularized and have little central necrosis, which may explain the low ctDNA concentration in the blood. To raise the likelihood of detecting these rare ctDNA fragments, many biomarkers can be screened at the same time (Elazezy and Joosse, 2018), however this raises the test complexity and price. The diagnostic power to detect tumors can be increased by combining multi-analyte modes of detection into a single test, such as the CancerSEEK test which combines sequencing ctDNA with detection of serum protein biomarkers (Cohen et al., 2017; Cohen et al., 2018). Another option is to examine ctDNA in alternate clinical specimens, e.g. in urine to diagnose bladder or prostate cancer, sputum for lung cancer, cerebrospinal fluid for glioma, and stool for CRC.

In the instance of CRC, an early stage tumor that sheds little ctDNA into the bloodstream may cause bleeding into the bowel and the FOBT or FIT will detect the hemoglobin resulting from this bleeding (Symonds et al., 2016). Conversely, late stage cancers might be more readily detected *via* the blood than stool (Ahlquist et al., 2012) and there is some evidence that people perceive a DNA-based stool test as preferable over FOBT (Schroy and Heeren, 2005) and a blood-based ctDNA test over a stool based test (Osborne et al., 2012; Adler et al., 2014). A comparison of the sensitivity of FIT and DNA-based tests to detect advanced precancerous lesions, early- and late-stage cancer is presented on **Table 2**. For CRC, a three-protein ELISA panel has been developed that has higher sensitivity and specificity for early stage I-II disease than the FOBT (Fung et al., 2015). This work has been translated into a company (https://www.rhythmbio.com/). 

**Table 2:** Comparison of commercially available assays for CRC.

	OC-SENSOR^®^	ColoGuard^®^	Epi proColon^®^	Epi proColon^®^	IKZF1/BCAT1
Assay	Fecal immunochemical test (FIT); 100 µg Hb/g	*KRAS* mutations, methylated *NDRG4*, *BMP3* and hemoglobin	Methylated *SEPT9*	Methylated *SEPT9*	Methylated *BCAT1* and/or *IKZF1*
Biosample	Stool	Stool	Blood	Blood	Blood
Study cohort size	9989 (65 tumors)	9989 (65 tumors)	1544 (44 tumors)#	1510 (53 tumors)#	2101 (85 tumors)
Specificity	94.9%	86.6%	80.0%	91.5%	93.8%
Sensitivity	73.8%	92.3%	68.2%	48.2%	65.9%
Advanced precancerous lesions*	23.8%	42.4%	21.6%	11.2%	6.2%
Stage I	65.5%	89.7%	41.1%	35.0%	37.9%
Stage II	76.2%	100.0%	83.3%	63.0%	69.0%
Stage III	90.0%	90.0%	80.0%	46.0%	72.5%
Stage IV	75.0%	75.0%	100.0%	77.4%	93.8%
Data reference	(Imperiale et al., 2014)	(Imperiale et al., 2014)	(Potter et al., 2014)	(Church et al., 2014)	(Pedersen et al., 2015b)

*Defined as advanced adenomas and sessile serrated polyps measuring 1 cm or more

#Standardized estimates

Liquid biopsy tests and traditional medical imaging can also be combined as they offer complementary means to detect and monitor cancer. While ctDNA tests are less expensive than imaging, and can characterize a tumor and potentially earlier detection of recurrence (Ulrich and Paweletz, 2018), these tests do not routinely identify the location of the tumor. The ability for medical imaging to identify the location of tumor(s) is particularly important pre-surgery and in metastatic disease. In the context of lung cancer screening, liquid biopsy tests have the advantage that they do not present high-risk populations (typically older smokers) with a lung radiation dose. In the primary diagnosis setting, ctDNA tests can be used as triage diagnostics after a scan, when a low-dose CT scan or mammography, for example, reveals an indeterminate mass. With sufficient specificity and sensitivity, ctDNA tests may replace riskier biopsy procedures.

### Product-Market Fit

With the increasing rise in chronic illness, aging populations, and climbing national healthcare expenditure, governments are increasingly looking at costs per relevant clinical outcome (Anderson and Frogner, 2008; Anderson et al., 2014). Fee-for-service payment models which reward volume will be replaced by quality metrics which value health outcomes achieved per dollar spent (Conway, 2009). Furthermore, the rise in precision therapies in oncology is creating the opportunity for more tailored treatments. As such, global healthcare in the 21st century is characterized by evidence-based medicine, patient-centered care, and cost effectiveness (Bae, 2015). In determining the market value of an *in vitro* diagnostic (IVD), technology investors and healthcare payers need to be provided with the appropriate evidence. It follows that the perceived value of an IVD is proportional to the quality of the evidence.

A well-tested case around product-market fit is useful for defining the clinical gap, who might be willing to order the IVD, who are the payers and if the proposed technological solution is a match for the identified marketplace. Factors to consider are price, assay time and performance metrics like sensitivity and specificity as well as logistics and the potential to meet market demand and expectations. For example, in a centralized lab model, one needs to consider how the analyte(s) are transported and the sample conditions required, and for assays with large potential markets, such as the primary diagnosis of common cancers, how the IVD be simplified, sequenced, and automated to scale to potentially huge volumes of tests per year. The 2010 review on the development of Epi proColon (Payne, 2010) provides an informative narrative on the development and translation of epigenetic diagnostics. From experience, Payne emphasizes that the platform and degree of test automation must be considered early in development and that the test should be robust to detect very low numbers of target molecules in a high background of non-target DNA.

Diagnostics should not just have technical or classification merit but must directly inform clinical decision making in a timely manner. Assays which define prognostic risk or estimate survival can assist in clinical decision-making regarding prescription of a more aggressive protocol or second-line therapy in poor prognosis cases, or in cases of likely predicted recurrence of metastatic disease, an increase in patient surveillance. While the latest molecular technologies can offer benefits to IVD performance metrics, IVDs depending on new technologies can be expensive to implement, automate, and regulate. More expensive IVDs are potentially a better fit for clinical decisions with large financial costs or health risks, such as a decision to administer a second-line therapy or to undertake a significant surgical procedure.

The utility of a primary diagnosis IVD should not just be considered in terms of the number of additional cancers detected over standard care, but also the costs and risks, both for the patient and to the healthcare system, for reporting false positive results. The costs and risks for each tumor type are contextualized by the incidence rate and available follow-up procedures. The true positive rate, known as positive predictive value (PPV), can be increased by targeting the clinical translation to higher risk sub-populations, such as smokers for lung cancer and BRCA mutation carriers for ovarian cancer, but even then, issues remain (Pearce et al., 2015). The problem of unnecessary procedures and patient psychological harm is very real in screening programs. For example, using low-dose CT for lung cancer screening, results from The National Lung Screening Trial revealed that 80.5% of cancer-free participants experienced unnecessary follow-up imaging studies, with 2.2% of participants having an invasive bronchoscopy procedure and 1.3% unnecessary surgery. Another screening study using cancer antigen 125 (CA-125) found that for each ovarian and peritoneal cancer detected by screening, an additional two women had false-positive surgery with a surgical complication rate of 3.1% (Jacobs et al., 2016).

Product-market fit needs to be considered early in the diagnostic development process. The identification of the prospective markets, clinically relevant patient group(s) and what clinical decisions happen after a positive test result should inform considerations around price, required turnaround time and minimal sensitivity and specificity metrics. The market size informs scale considerations and the biosample collection procedure, the need for ambient or cold chain transport logistics. All these parameters collectively inform the design of the diagnostic assay.

### Pre-Analytic Conditions

Before the collection of clinical samples, the pre-analytic conditions for how tumor biopsies, blood, or other biosamples will be prepared and stored for later analysis require consideration, including quality control for sample integrity (e.g. cell lysis or nucleic acid degradation). It is commonplace for tumor tissue sections to be stored in a fixative. By necessity, diagnostic tests utilizing tissue sections need to be robust to analyze potentially heavily degraded DNA in formalin fixed paraffin-embedded (FFPE) samples. As the liquid biopsy diagnostics area matures toward increased clinical translation, there is a strong focus on controlling for pre-analytical variables. Guidelines are now coalescing around the optimal preanalytical conditions for analyzing cfDNA (Meddeb et al., 2019) and two large consortia have formed to standardize pre-analytical steps and downstream protocols. The CANCER-ID European Public-Private-Partnership (www.cancer-id.eu) commenced at the start of 2015 and has 36 partners from 13 countries with aims to establish standard protocols for clinical validation of blood-based biomarkers. The USA-based Blood Profiling Atlas in Cancer (BloodPAC; www.bloodpac.org) consortium formed in 2016 is aggregating, harmonizing, and making freely available data from CTC, ctDNA, protein and exosome assays, and the associated clinical data and biosample collection protocols.

### Suitable Tissue and Analytes

The stability of epigenetic marks on DNA means there a few limitations on possible analytes with almost all tissues useful for designing DNA methylation-based diagnostics.

#### Blood

Blood represents a rich source of information on tumor biology and is usually the tissue of choice for ctDNA studies. DNA methylation can be assayed easily using existing methods. There is potential for other epigenetic data to be determined from ctDNA, such as nucleosome positioning and gene activity. Using sequencing approaches, ctDNA fragment ends can be used to estimate genomic activity (Snyder et al., 2016) and predict gene expression (Ulz et al., 2016) without biopsying the tumor itself. While this is a very early area of research these findings open the window to detailed assessments of intra-tumoral biology without access to tumor tissue and without dependence on just one epigenetic mark (i.e. DNA methylation).

The use of ctDNA in clinical settings does have a set of known caveats, in particular, low yields of DNA and the level of contaminating DNA from other cells. The bulk of cfDNA found in blood derives from nucleated blood cells, with a proportion from vascular endothelial cells and liver (Moss et al., 2018). Special consideration must be taken in handling blood samples in the clinical setting, as white blood cell lysis can produce large quantities of fragmented DNA. Typically, ctDNA represents only a very small fraction of total cfDNA, so inappropriate handling of blood samples may result in near complete loss of measurable signal. This risk can be abrogated through the use of careful blood processing techniques or specialized cfDNA collection tubes which stabilize white blood cells (Meddeb et al., 2019). Examples include PAXgene^®^ Blood ccfDNA Tube (Qiagen), Cell-Free DNA Collection Tube (Roche), cf-DNA/cf-RNA Preservative Tube (Norgen Biotek), and Cell-Free DNA BCT® (Streck).

#### Urine

Sources of cfDNA in urine can be categorized into three sources: pre-renal that can be mostly attributed to blood cells (from the systemic circulation), renal, and post-renal from the bladder urothelium. The median relative contributions of these three tissues are around 52%, 32%, and 5%, respectively. These values do vary largely across patient urine samples, but the ranked order is consistent (Cheng et al., 2017). Compared to blood cfDNA testing, urine cfDNA has two advantages; firstly, it is far easier and cheaper to obtain urine than blood, making urine an ideal biofluid in resource-limited settings (Lawn et al., 2012). Secondly, urine is thought to be a more sensitive alternative for early detection or monitoring recurrence of cancers in the genitourinary tract (Lin et al., 2017). Presently, none of the registered cancer IVDs are based purely on urinary cfDNA. One major reason is because the workflow in preserving urine cfDNA has yet to be standardized. The activity of DNase I in urine, relative to serum, is around 100-fold higher (Bryzgunova and Laktionov, 2015); as such, the half-life of urine cfDNA at body temperature is around 2.6–5.1 h (Cheng et al., 2017).

For clinical purposes, methods that stabilize urine cfDNA and prevent the lysis of nucleated cells are imperative to ease end-user collection. Some products addressing this unmet need have entered the market. These preservatives tend to be colored liquids (to provide visual indication for their addition), or as a dried coating lining the collection container. Examples include Urine Preservation (Norgen Biotek), Cell-Free DNA Urine Preserve (Streck), Quick-DNA Urine Kit (Zymo Research), and NextCollect™ (Novogene). For research purposes, there are kits designed specifically for urine cfDNA, but across the kits, the extracted DNA displays significantly different yields and size profiles (Diefenbach et al., 2018; Streleckiene et al., 2018).

As isolating urine cfDNA remains a technically challenging problem, current biomarker discovery efforts are mostly based on the cellular fraction of the collected urine. Compared to blood, practical use of urine markers in detecting or monitoring cancer is limited. One contributing factor is that cut-offs or thresholds derived from clinical studies tend to be specific to the study despite a focus on the same marker; the lack of standardized methodology also leads to different definitions of optimality. Binary thresholds resulting from differing definitions are problematic, more so for patients close to the cut-off point (Lotan et al., 2010). Currently marketed tests address this limitation by relying on a panel of biomarkers (Gallioli et al., 2019), or constrain themselves to recurrence monitoring.

#### Stool

Analysis of fecal material is useful for a range of bowel conditions, e.g. efficiency of digestion, leaky gut syndrome, inflammatory bowel disease, dysbiosis, acute infections, and CRC (Siddiqui et al., 2017). Stool testing for CRC is widely used and robust collection regimes are well established with home-based collection kits routinely used. Test kits have a stabilization agent as this is critical for maximizing the performance of fecal DNA-based tests (Olson et al., 2005; Nechvatal et al., 2008) and stool contains polymerase chain reaction (PCR) inhibitors, which need to be removed (Flekna et al., 2007). The fraction of human epithelial cell origin DNA in stool is small compared to total bacterial DNA, so a PCR diagnostic assay must also be robust to this background (Nechvatal et al., 2008).

#### Airway

Studies have demonstrated that methylated DNA can be detected within respiratory derived biological samples, specifically sputum (Hulbert et al., 2017), bronchoalveolar lavage (Um et al., 2017), nasal washing/brushing (Yang et al., 2017a; Nino et al., 2018), and exhaled breath condensate (EBC) (Xiao et al., 2014). Not surprisingly, the majority of the literature has focused on the role of this methylated DNA in lung associated pathologies such as asthma, cystic fibrosis, and lung cancer (Konstantinidi et al., 2015).

After a radiological procedure highlights an indeterminate lung mass, a reasonable first step in the investigation is the cytological analysis of sputum to detect lung cancer associated cells. This has a clinical sensitivity of 66%. Further follow-up tests with higher sensitivity are likely required, such as the biopsy of suspected lung nodules (90% sensitivity), but this is a highly invasive and risky procedure, with a 15% chance of collapsing a lung (pneumothorax) (Rivera et al., 2013). Detection of methylated ctDNA is presenting as a viable alternative to cytology of sputum. In a large cohort of lung cancer patients, it was demonstrated that measuring the methylation pattern of eight genes had a lung cancer prediction accuracy of 82%-86%, and a negative predictive value (NPV) from 88% to 94% to rule out cancer (Leng et al., 2017). Another study demonstrated that using the methylation status of genes *TAC1*, *HOXA17*, and *SOX17* in sputum had a sensitivity of 93% to detect lung cancer (Hulbert et al., 2017). These studies show that DNA methylation detection in sputum has greater sensitivity than sputum cytology. However, a major problem is that there is no standardization of sputum acquisition and handling so pre-analytical variables remain a major challenge to translation (Rivera et al., 2013).

Bronchoalveolar lavage is a process where bronchoscopy is used to locate the lung lesion, which is subsequently washed (lavage) with 10–20 ml of isotonic saline and collected for analysis. For easily visible and accessible central lesions, forcep biopsies of the lesions are performed (74% sensitivity) followed by bronchoalveolar lavage (48% sensitivity). However, the sensitivity is lower for peripheral lung lesions as they are difficult to locate and visualize, with a sensitivity of 57% and 43% for transbronchial biopsies and bronchoalveolar lavage, respectively (Rivera et al., 2013). While cytology analysis is typically performed on bronchoalveolar lavage (Carvalho et al., 2017) there is also opportunity to use the lavage fluid for liquid biopsy. Methylated *SHOX2* and *RASSF1A* gene promoters were detected in lavage fluid from 322 patients with a sensitivity of 81% for lung cancer detection (Zhang et al., 2017). Other studies have also revealed high sensitivity for lung cancer detection using bronchoalveolar lavage fluid, with 75% and 78% for *PCDHGA12* (Jeong et al., 2018) and *SHOX2* (Dietrich et al., 2012) methylated DNA, respectively. However, these studies use different methodologies to process the lavage samples, so cannot be directly compared.

The collection of EBC is a novel non-invasive measurement method for lung cancer detection. A portable FDA approved device exists for EBC (RTube™ by Respiratory Research, Inc.), however, there are several caveats with using EBC collection for diagnostic purposes. These include the dilution of analytes in the breath condensate and the contamination with DNA from ambient air, saliva, and the nasal epithelium (Horvath et al., 2017; Koc et al., 2019). Furthermore, normalizing for varying levels of condensation arising from different collection methods is a well-known issue (Horvath et al., 2017). While there are several challenges to overcome in developing an IVD, the non-invasive nature of EBC compared to bronchoalveolar lavage makes EBC an attractive biological sample.

### Technology

#### Overview

Bisulfite treatment is the gold standard method for mapping methylated cytosines in DNA and was developed by Australian scientists from the CSIRO and Kanematsu Laboratories in Sydney (Frommer et al., 1992; Clark et al., 1994). With this method, sodium bisulfite is used to convert cytosine residues to uracil residues in single-stranded DNA, under conditions whereby 5-methylcytosine (5mC) remains non-reactive. The 5-hydroxymethylcytosine (5-hmC) epigenetic mark, which is mostly confined to embryonic stem cells and to an extent brain and liver, is indistinguishable from 5mC using bisulfite conversion (Huang et al., 2010). Alternatives to bisulfite treatment are to use enzymes sensitive (or specific) to DNA methylation within their cleavage site or affinity capture using a binding protein or antibody. Bisulfite-treatment can be coupled with multiplexed probe-based detection. Methods which selectively determine the presence of methylated DNA are a good fit for liquid biopsy applications, whereas methods estimating the fraction of DNA methylated at a CpG site (often called the beta-value), are better suited for examining tissue. A brief description and classification of commonly used methods is presented on **Table 3**.

**Table 3:** Summarised methods for the detection of DNA methylation in liquid biopsy.

Method name	Class*	Sub-class	Bisulfite-based	Description	Citation
Whole Genome Bisulfite Sequencing (WGBS)	GW	High-throughput sequencing	Y	Various approaches for the tagging and sequencing of bisulfite converted DNA. Adapter tagging can be done before or after conversion. Different approaches may introduce biases.	(Lister et al., 2009)
Bisulfite Sanger Sequencing (Bis-Seq)	TGT	Bisulfite conversion specific amplification	Y	Bisulfite-treated genomic DNA subjected to amplification with conversion-specific PCR primers. Primers are unbiased and contain no CpGs	(Frommer et al., 1992)
Nested PCR	TGT	Bisulfite conversion specific amplification	Y	Bisulfite-treated genomic DNA subjected to amplification with conversion-specific PCR primers, followed by secondary PCR with primers targets within the first PCR fragment to enhance specificity or sensitivity.	(Herman et al., 1996)
Methylation-specific PCR	TGT	Bisulfite conversion specific amplification	Y	Bisulfite-treated DNA subjected to amplification. PCR primers intentionally biased by including multiple CpGs in binding sites.	(Herman et al., 1996)
MethylLight	TGT	Fluorescence probe PCR	Y	Conversion or Methylation specific PCR with the addition of a TaqMan probe. Methylation specificity obtained by including CpGs in the primers, probe or both.	(Eads et al., 2000)
Quantitative Allele-specific Real-time Target and Signal amplification (QuARTS)	TGT	Fluorescence probe PCR	Y	Bisulfite-treated genomic DNA subjected to PCR with probes targeting alternate methylation states. Probe fluorescence activated by an additional oligo binding immediately upstream.	(Zou et al., 2012)
HeavyMethyl	TGT	PCR with blocker	Y	Competitive inhibition of PCR using primers combined with a blocker oligo that target alternate methylation states.	(Cottrell et al., 2004)
Cold-PCR	TGT	Preferential denaturation temperature PCR	N	The first few cycles are conventional PCR. Subsequent cycles use a lower denaturation temperature to enrich for DNA molecules that contain mismatches, which occur if there are mutant DNA sequences in the sample.	(Milbury et al., 2011; Castellanos-Rizaldos et al., 2014)
Ice-COLD-PCR	TGT	Preferential denaturation temperature PCR	N	The same as cold-PCR but with the addition of a further blocker oligonucleotide to inhibit amplification of unwanted targets.	(Milbury et al., 2011; Mauger et al., 2018)
High-resolution melt (HRM) curve analysis	TGT	Melt-curve analysis	Y	Following traditional PCR with an intercalating dye (e.g. SYBR green), the PCR product is gradually warmed until the DNA strands denature (melt) apart. DNA melting is detectable by shifts in the level of fluorescent signal over time.	(Wittwer, 2003; Wojdacz and Dobrovic, 2007)
Bis-seq (pyrosequencing)	TGT	Bisulfite conversion specific amplification	Y	“Sequencing by synthesis” method. Sequence readout is obtained by detecting pyrophosphate released during base incorporation during synthesis of the complementary DNA to the target fragment.	(Ronaghi, 1998; Fraga and Esteller, 2002)
EpiTYPER	TGT	Mass-spectrometry	Y	Utilizes mass spectrometry to accurately measure the methylation of PCR-derived amplicons. DNA is converted and amplified by PCR. Incorporation of C/G or T/A bases (methylated or unmethylated) during amplification leads to measurable shifts in molecular weight.	(Ehrich et al., 2005)
Reduced representation bisulfite sequencing (RRBS)	RGW	Enzymatic digest	Y	Genomic DNA digested with methylation-insensitive restriction enzyme (with CpG in the recognition site), followed by size selection prior to bisulfite conversion.	(Meissner et al., 2005)
Combined Bisulfite Restriction Analysis (COBRA)	RGW	Enzymatic digest	Y	Bisulfite-treated genomic DNA is subjected to methylation-insensitive restriction enzyme digest targeting the unconverted amplicon (i.e. originally methylated). Ratio of digested fragments to total fragments correlates with methylation level.	(Xiong and Laird, 1997)
Digital Restriction Enzyme Analysis of Methylation (DREAM)	RGW	Enzymatic digest	N	Methylation specific restriction enzyme (MRSE) variant. DNA digested with two enzymes, one methylation sensitive and one not. Methylation readout based on ratio of cutting.	(Jelinek and Madzo, 2016)
Methylation-sensitive restriction enzyme (MSRE) + qPCR	TGT	Enzymatic digest	N	Unconverted DNA is digested with MRSE. Quantitative PCR is used to establish the efficiency of digestion, which indicates the level of methylation at the target site.	(Hashimoto et al., 2007)
Helper-dependent chain reaction (HDCR)	TGT	Enzymatic digest	N	Genomic DNA digested with methylation dependent restriction enzyme such as GlaI. Gene-specific sequence fragments are tagged with “helper” oligos, while “driver” oligos maintains preferential amplification of tagged fragments.	(Rand et al., 2013)
End-specific PCR (ES-PCR)	TGT	Enzymatic digest	N	MRSE variant for detecting unmethylated sequences. DNA is digested with a methylation sensitive enzyme, then specialized oligos are used to add priming sites to the target sequence. Highly useful method for targeting repetitive sequences that are difficult to assay by other methods.	(Rand and Molloy, 2010)
MeDIP	RGW	Affinity capture	N	DNA capture using antibody specific to methylated cytosine. Captured DNA suitable of PCR, array and sequencing based methods.	(Weber et al., 2005)
Various methyl-CpG binding domain (MDB)-based assays	RGW	Affinity capture	N	DNA capture using methylated DNA binding protein (MBD2). Use of salts during elution from MBD can facilitate fractionation on methylation level. Captured DNA suitable of PCR, array and sequencing based methods.	(De Meyer et al., 2013; Aberg et al., 2015)
SuBLiME	RGW	Affinity capture	Y	Biotinylated bases are incorporated into DNA fragments using a PCR-like approach following bisulfite conversion. Biotinylated fragments are captured to enrich for methylated targets. Can be performed in a targeted or genome-wide method.	(Ross et al., 2013)
Bisulfite Specific Padlock Probes (BSPP)	RGW	Molecular inversion probes	Y	BSPP utilizes bisulfite converted DNA and specialized DNA probes. Probes bind two sites in target sequences to form circular DNA structures that can be amplified and sequenced.	(Diep et al., 2012)
Infinium HumanMethylation	RGW	Infinium assay	Y	Bisulfite treated DNA is hybridized to the BeadArray chip, detection using single-base extension and fluorescence ratio between converted and unconverted probes.	(Bibikova et al., 2011)

*GW, genome-wide; RGW, representative genome-wide; TGT, targeted.

#### Bisulfite-Treatment

Bisulfite treatment of DNA for diagnostic purposes is not without issues. Foremost, is the significant loss of material due to the harsh chemical and temperature conditions involved (Grunau et al., 2001). This loss reduces sensitivity to detect cancers, especially those releasing low levels of ctDNA. In addition, there is a loss in genome complexity due to the large reduction in the prevalence of cytosine bases in converted DNA, resulting in a largely pseudo-three base genome. Careful PCR primer design is required to specifically amplify rare target molecules in an overwhelming off-target background, such as with a typical methylation-based ctDNA assay.

Bisulfite-treated DNA is typically amplified using conversion-specific PCR (CSP) or methylation-specific PCR (MSP) primers. With CSP, primers are designed to amplify bisulfite-converted DNA regardless of methylation state; while with MSP the primers target unconverted cytosines, such that only methylated DNA is amplified (Herman et al., 1996). To achieve amplification specificity with bisulfite-treated template DNA, nested PCR is sometimes used. However, this is not an optimal fit with an IVD due to exposure of amplified DNA from the first round of PCR into the clinical lab environment. Bisulfite treatment of DNA can be combined with NGS. The entire methylome can be sequenced *via* whole genome bisulfite sequencing (WGBS), or regions targeted by sequencing CSP amplicons. Genome regions can also be targeted using a technique like reduced representation bisulfite sequencing (RRBS), where DNA is digested with a methylation-insensitive restriction enzyme (with CpG in the recognition site), followed by size selection prior to bisulfite conversion (Meissner et al., 2005).

MSP is used to amplify cancer DNA from hypermethylated promoters. With ctDNA assays, the cancer-originating DNA is rare compared to background off-target DNA, so additional measures are often needed such that the PCR assay remains specific even after the large number of amplification cycles needed to observe rare ctDNA. The MethyLight assay is a quantitative MSP with the addition of a TaqMan-based fluorescent probe. It is sensitive for methylation levels as low as 0.01% and has good reproducibility (Eads et al., 2000). The Quantitative Allele-Specific Real-time Target and Signal amplification (QuARTS) method also employs a probe but in addition incorporates a 5´ DNA flap, a flap endonuclease and fluorescence resonance energy transfer (FRET) chemistry for detection of the cleaved products (Zou et al., 2012). The HeavyMethyl method is a quantitative CSP amplification which adds a blocker oligonucleotide that competes for binding across the primer sites to unmethylated DNA, thus preventing efficient amplification of unmethylated DNA (Cottrell et al., 2004).

Other properties of bisulfite-treated DNA can be used to selectively amplify the target molecule, such as preferential amplification using denaturation temperature. This family of methods includes co-amplification at lower denaturation temperature PCR (COLD-PCR) (Milbury et al., 2011; Castellanos-Rizaldos et al., 2014) and bisulfite differential denaturation PCR (Rand et al., 2006), where the basic principle is to select a critical temperature in the PCR to selectively denature unmethylated genomic regions in the presence of an excess of methylated DNA molecules. The methylation-sensitive high-resolution melting (MS-HRM) method uses the difference in melting temperature between methylated versus unmethylated product after a CSP reaction to quantify methylation (Wojdacz and Dobrovic, 2007). Methylation may also be quantified using a PyroMark pyrosequencer (Qiagen) or the EpiTYPER^®^ mass spectrophotometry instruments (Agena Biosciences). Both approaches can detect small changes in methylation.

#### Enzyme Cutting

Enzyme-based methods offer an alternative to bisulfite-treatment and are not subject to the same losses of material. The disadvantages are that assayed regions must overlap loci of interest and that incomplete digestion can confound interpretation of the results. Methylation-sensitive restriction enzyme (MSRE) cutting can be coupled with quantitative PCR to estimate DNA methylation, with more product proportional to more methylation at the cut site(s) within the amplicon (Hashimoto et al., 2007). Conversely, a methylation-dependent enzyme such as GlaI can be used to selectively cut only methylated DNA. The selective amplification of DNA with ends cut by GlaI is used in the end-specific PCR (ES-PCR) and helper-dependent chain reaction (HDCR) techniques (Rand and Molloy, 2010; Rand et al., 2013). The Combined Bisulfite Restriction Analysis (COBRA) method is a hybrid which involves cutting DNA that has been first bisulfite-treated and PCR amplified (Xiong and Laird, 1997).

The Digital Restriction Enzyme Analysis of Methylation (DREAM) is a method for mapping DNA methylation levels at a specific set of CpG sites that are contained within the recognition sequence, 5’-CCCGGG-3’ for two restriction enzymes, SmaI and XmaI (Jelinek et al., 2012). It relies on the differential sensitivity of the two enzymes to methylation at the central CpG site and their different modes of cutting. Cutting by SmaI is blocked by methylation of the central CpG site, while XmaI cuts whether the CpG site is methylated or not. Thus, methylated sites are scored indirectly as those 5’-CCCGGG sites that are not cut by SmaI.

#### Affinity Capture

Affinity capture techniques are used to enrich methylated DNA from the overall DNA population. This is usually accomplished by antibody immunoprecipitation methods or with methyl-CpG binding domain (MDB) proteins and there are modifications to the protocol that also enable hydroxymethylation capture (Thomson et al., 2013). Input genomic DNA can be sonicated or enzymatically digested prior to capture and purification, often *via* magnetic beads. Eluted DNA is usually then used as input for the generation of NGS libraries, but also suitable for analysis with microarrays or PCR-based methods. The different variants of this methodological principle result in widely different patterns of the distribution of DNA methylation enrichment (De Meyer et al., 2013; Aberg et al., 2015). An alternative affinity capture technique utilizes the incorporation of biotinylated cytosines during amplification of bisulfite-treated sheared or digested genomic DNA fragments followed by affinity capture using streptavidin-coupled magnetic beads (Ross et al., 2013).

#### Multiplexed Probe-Based Detection

The Infinium Methylation Assay detects cytosine methylation at CpG dinucleotides using single-base extension of two site-specific probes, one each for the methylated and unmethylated locus in a highly multiplexed reaction on bisulfite-converted genomic DNA. The level of methylation for the interrogated locus can be determined by calculating the ratio of the fluorescent signals from the methylated vs. unmethylated sites. This is by far the most widely used “genome-wide” DNA methylation analysis platform with significant amounts of public data available. The bioinformatics analysis pipelines for this platform are also mature. The currently available third iteration of this platform is the Infinium MethylationEPIC BeadChip, which interrogates 863,904 CpG sites.

Padlock probes are single stranded DNA molecules with two segments complementary to the target DNA connected by a linker sequence, which are hybridized to the DNA target to become circularized (Nilsson et al., 1994). Molecular Inversion Probes (MIP) are derivatives of padlock probes, although they contain a gap in the target sequence, which provides for greater flexibility. These probes can be used for various forms of genomic partitioning, single nucleotide polymorphism (SNP) genotyping, or copy-number variation detection. Bisulfite padlock probes (BSPP) are an adaptation for the analysis of DNA methylation (Ball et al., 2009; Deng et al., 2009; Diep et al., 2012), where padlock probes are hybridized to bisulfite-treated DNA and subsequently interrogated using NGS.

## Existing Registered Assays

The existing DNA methylation-based ctDNA IVDs with FDA Premarket Approval (PMA) or offered as LDT or European union CE-IVDs are summarized in **Table 1**. A description of these registered tests and upcoming tests on the path to registration follows.

### Bladder Cancer

Approximately 70% of bladder cancer cases are non-muscle-invasive (NMIBC). Lifelong post-operative surveillance is essential due to high recurrence rates (50%-70% patients experience recurrence within 5 years), and a moderate chance of disease progression to muscle invasion (10%-15%) (Tilki et al., 2011). The gold standard for diagnosis is cystoscopy and cytology; urinary tests have yet to achieve comparable specificity or sensitivity. However, monitoring for recurrence could be safer and cost effective if the non-invasive test had a high NPV (Witjes et al., 2018).

Bladder EpiCheck^®^ (Nucleix) is a urine assay for NMIBC based on 15 proprietary methylation biomarkers. DNA is extracted from centrifuged cell pellets from 10+ ml of patients’ urine, and subjected to methylation-sensitive restriction enzyme digestion before quantitative PCR (qPCR) amplification. The quantitative results are summarized as an EpiScore ranging from 0 to 100 (where scores ≥ 60 are considered positive for recurrence). This test has a reported NPV of 95%-97% and is currently available as a CE-IVD in the EU (Wasserstrom et al., 2016; Witjes et al., 2018; D’Andrea et al., 2019).

Similarly, Bladder CARE™ (Pangea Laboratory) is a urine assay for NMIBC recurrence based on the hypermethylation of a proprietary three-gene panel, likely *SOX1*, *IRAK3*, and methylated LINE1 (Su et al., 2014). According to unpublished material released by the company, the urine sample (∼5 ml) is first mixed in a 1:3 ratio with a stabilization buffer prior to shipment to their clinical lab where DNA is harvested from centrifuged cell pellets, digested with methylation-sensitive restriction enzymes, then amplified with qPCR. Results are summarized as three calls: negative, high-risk, or positive. This LDT currently targets the bladder cancer recurrence market, but promotional materials raise the possibility of early detection due to the high reported PPV and NPV of the test (89% and 92%, respectively).

Hematuria (blood in urine) can be an early sign of bladder cancer, where 3%-28% of patients with hematuria are diagnosed with bladder cancer. AssureMDx™ for Bladder Cancer (MDxHealth) is a urine assay that excludes bladder cancer diagnosis based on a negative result (99% NPV), leading to 77% reduction in diagnostic cystoscopies, resulting in lower diagnostic costs and reduced patient burden (van Kessel et al., 2017). DNA from cells in the urine samples are subjected to a methylation specific PCR targeting three genes (*OTX1*, *ONECUT2*, and *TWIST1*). In addition, the mutation status of three other genes (*FGFR3*, *TERT*, and *HRAS*) provided additional support for the predictive model (Su et al., 2014; van Kessel et al., 2016; van Kessel et al., 2017). This product is currently available as an LDT in the USA.

A promising candidate test for patients presenting with hematuria is UroMark (University College, London), currently in validation studies in the UK. The initial study demonstrated high PPV and NPV (100% and 97%, respectively) (Feber et al., 2017). This test detects the methylation status of 150 loci across the genome, which is obtained from subjecting cell pellets from urine samples to a microdroplet-based PCR amplification of bisulfite-converted DNA.

### Breast Cancer

Breast cancer is a highly heterogeneous disease and molecular subtyping has proven effective in reducing mortality. Breast cancer subtypes and treatments are traditionally determined using histopathology for key hormone receptors that are also the targets of most common frontline therapies. Recent IVDs utilizing gene expression and mutational profiles aim to stratify patients into risk/treatment groups (e.g. PAM50/Prosigna, OncotypeDX, and Endopredict). These methods use traditional tissue biopsies and do not make use of DNA methylation. Despite the success of molecular testing in breast tumors, current DNA methylation-based assays and liquid biopsy offerings in breast cancer are sparse and no methylation-based ctDNA assays are available. Current DNA methylation-based offerings are limited to the therascreen^®^ PITX2 RQG test developed by Qiagen/Therawis which is available as a prognostic/predictive CE-IVD in the EU.

Qiagen’s therascreen^®^ PITX2 RGQ PCR Kit is a qPCR-based assay that determines the ratio of methylated to unmethylated DNA content in tumor histology sections, where percent methylation ratio (PMR) is indicative of overall survival and patient outcome when anthracyclines are combined with standard therapy (Maier et al., 2007). Anthracyclines carry serious side that may limit treatment (Volkova and Russell, 2012) and patients with less aggressive tumors subtypes or other contraindications may be adequately treated with standard approaches (Turner et al., 2015). By using the therascreen^®^ PITX2 assay, the risk of over-treatment can be minimized without risk to patient outcomes. However, the therascreen^®^ test is limited to estrogen receptor-positive, node-negative tumors only. The more aggressive and/or difficult to treat HER2-positive and triple-negative subtypes or tumors with lymph node involvement do not benefit from this assay.

### Cervical Cancer

The screening and detection of cervical cancer has been transformed by the relatively recent discovery of the role of Human Papilloma Virus (HPV) in the initiation and progression of this disease. Traditional cytological screening has now been displaced by modern molecular methods that target HPV. These new approaches are both cheaper and more effective at identifying at risk women, even when screening intervals are increased (Brotherton et al., 2016). The development of the highly successful vaccine against HPV will have a continuing disruptive impact on cervical cancer screening with HPV incidence in young women trending toward zero in nations with effective vaccination programs (Read et al., 2011; Ali et al., 2013; Brotherton et al., 2016).

Unsurprisingly, epigenetic diagnostics available in the market have positioned themselves as triage tests following positive HPV findings. Three competing tests exist in the marketplace, QIAsure (Qiagen), GynTect^®^ (Oncgnostics GmbH), and the CONFIDENCE assay (Neumann Diagnostics). However, the DNA methylation component of the Neumann assay is currently awaiting full certification. All three tests utilize liquid samples from cervical scrapings/smears with minor differences in methodology. GynTect offers a slightly more streamlined protocol when compared to QIAsure, with no dedicated DNA extraction step. QIAsure offers an alternative convenience for patients, in the fact that it offers a process for both physician and self-collected cervical samples without loss of sensitivity (De Strooper et al., 2016) whereas GynTect and CONFIDENCE are limited to physician collected samples only. Target genes are also another source of difference, with QIAsure targeting the promoters of tumor suppressor genes *FAM19A4* and *hsa-mir124-2* and another non-specific positive control. GynTect targets a larger number of genes including *ASTN1*, *DLX1*, *ITGA4*, *RXFP3*, *SOX17*, and *ZNF671* plus two quality control regions. The CONFIDENCE assay targets the fewest sites, measuring methylation at the *POU4F3* gene and one other control region (*COL2A1*) (Kocsis et al., 2017).

The utility of these assays in triaging patients exists in the epigenetic biology of HPV-driven carcinogenesis. HPV detection on its own is not necessarily indicative of the likely presence of cancer, most infections will be benign, and those patients will require no further treatment. Malignant infections will trigger the expression of pro-oncogenic viral genes leading to the formation of the precursor lesion transforming cervical intraepithelial neoplasia (CIN). As CIN progresses from low to high grade (CIN1–3) there is a sequential build-up of DNA methylation aberrations across the genome. By targeting genes associated with high grade/risk CIN these tests can provide as surrogate for CIN grade that can be used to stratify patients into high/low risk groups. In terms of assay performance, QIAsure’s sensitivity of 70.5% for CIN3+ samples exceed GynTect’s 61.2%. However, GynTect does have a substantially improved specificity over QIAsure (94.6% and 67.8% for GynTect and QIAsure respectively (De Strooper et al., 2014; Schmitz et al., 2018). The predictive values of both tests are comparable, with NPV for both tests ∼90% although QIAsure’s reduced specificity does result in better PPV for GynTect. Ultimately, published data shows the two tests to be comparable in performance although ongoing trials may distinguish the two sometime in the future.

### Colorectal Cancer

For primary diagnosis of CRC two tests have progressed through to FDA PMA approval, the blood-based Epi proColon**^®^** (Epigenomics) and the stool-based Cologuard^®^ (Exact Sciences). Both tests are approved for patients ≥ 50 years of age and require follow colonoscopy for a definitive diagnosis. ColoSure™, a stool-based LDT for primary diagnosis test which detects methylated *VIM* (Ned et al., 2011) has been withdrawn from sale. More recently, COLVERA™, a blood-based test for the detection of CRC recurrence, has been distributed in the USA as an LDT since 2016.

Cologuard^®^ is a stool-based DNA test which consists of a regular FIT together with amplification of methylated *BMP3* and *NDRG4*, β-actin methylation control, and mutant *KRAS*. Cologuard^®^ has been tested in a large asymptomatic screening population consisting of 9,989 patients (Imperiale et al., 2014) and found to have sensitivity for CRC detection similar to that of colonoscopy, and superior sensitivity for advanced precancerous lesions and early stage cancer when compared to FIT (**Table 2**). However, the specificity is lower with Cologuard^®^ in comparison to FIT (**Table 2**). Cologuard^®^ was approved as a screening test for CRC by the FDA in 2014. Cologuard’s estimated market share after Q1 2019 is 4.6% and approximately a million tests were ordered in 2018. Exact Sciences is also submitting an application to expand Cologuard’s label to include the 45–49 age group in accordance with updated screening guidelines in the USA (American Cancer Society, 2019) to increase the test’s market opportunity. Together with researchers at the Mayo Clinic, Exact Sciences is also currently developing an updated version of the test with additional biomarkers.

Epi proColon^®^ has had less market traction than Cologuard^®^. Epi proColon^®^ detects the presence of methylated *SEPT9* in plasma; it has higher specificity than Cologuard^®^, but less than FIT and is less sensitive than both. Epi proColon^®^ is not recommended for routine screening of CRC, but is an alternative to patients, 50 years or older, with average risk for CRC, who decline other CRC screening such as FIT or screening colonoscopy.

After surgical resection and subsequent chemotherapy treatment for CRC, there is a 30%-50% chance that the disease will recur within 5 years. This is typically observed as distant metastases of the liver, lung, or locoregional areas (Duffy et al., 2003). Carcinoembryonic antigen (CEA) has historically been the only non-invasive biomarker in routine clinical practice for surveillance of disease recurrence. However, CEA has poor sensitivity (35% with 95% specificity) and blood CEA levels are not elevated in 58% of CRC patients (Goldstein and Mitchell, 2005). Although serial measurements of CEA are widely used in surveillance, there is variable agreement about what constitutes a clinically significant increase. The European Group on Tumour Markers (EGTM) guidelines guardedly define this as at least 30% over the previous value with increase to be followed by a second sample taken within 1 month and a confirmed trend investigated to detect or exclude malignancy (Duffy et al., 2003).

CSIRO co-developed the methylated two-gene (*IKZF1* and *BCAT1*) panel COLVERA™ liquid biopsy test with Clinical Genomics and the Flinders Centre for Innovation in Cancer (Mitchell et al., 2014; Mitchell et al., 2016). COLVERA™ has been available since 2016 in the USA as an LDT to detect residual disease post-surgical resection and for surveillance of recurrent CRC after primary treatment. COLVERA™ is informative with respect to completeness of surgical resection, risk of residual disease, and recurrence-free survival (Murray et al., 2018). It has double the sensitivity of CEA and should allow more judicious use of PET-CT (Young et al., 2016). The *IKZF1*, *BCAT1* marker pair also shows potential for primary diagnosis of CRC and has demonstrated better performance than the Epi proColon^®^ SEPT9 test (**Table 2**).

### Glioblastoma

*MGMT* (O6-methylguanine DNA methyltransferase) promoter methylation is inversely correlated with *MGMT* expression and patients’ response to the alkylating agent temozolomide (Esteller et al., 2000) with approximately 50% of grade IV glioma (usually glioblastoma, GBM) exhibiting *MGMT* promoter methylation (Wick et al., 2014). Multiple large-scale clinical studies have identified that patients having hypermethylation of the *MGMT* promoter region experience significant outcome benefit with temozolomide treatment (Hegi et al., 2005; Stupp et al., 2005). As such, testing for hypermethylated *MGMT* has entered standard care and management for patients with glioma and is a key factor for treatment strategy selection for GBM patients (Louis et al., 2016). To date, there is no consensus on the optimal method for detection of *MGMT* promoter methylation. MSP and pyrosequencing of bisulfite-treated DNA are the most common assay methods, with pyrosequencing likely displaying better performance compared to MSP (Havik et al., 2012). Some studies suggest PCR with HRM has better performance than MSP and pyrosequencing with regards to diagnostic accuracy and efficiency but further large-scale trials are needed to be validated (Switzeny et al., 2016).

There are several methylated *MGMT* IVDs on the market. The pyrosequencing-based therascreen^®^ MGMT Pyro^®^ (Qiagen) is a registered CE-IVD and can quantify four CpG sites in the first exon of *MGMT*. The Human MGMT Gene Methylation Detection Kit (Xiamen SpacegenCo) is also a CE-IVD and is based on Xiamen SpacegenCo’s proprietary PAP-ARMS^®^ technology which combines the pre-existing Amplification Refractory Mutation System (ARMS) approach with pyrophosphorolysis-activated polymerization (PAP), increasing specificity by preventing mismatched primer extension. LabCorp also offer PredictMDx™, an MSP-based test for detecting *MGMT* methylation in FFPE biopsies and licensed from MDxHealth.

Researchers in Heidelberg, Germany have developed an innovative methylation profiling tool for classification of central nervous system tumors based on the Illumina Human Methylation BeadChip data of 2,801 reference samples across adult and pediatric tumors (Capper et al., 2018). Classification by the tool resulted in the revision of the initial histopathological diagnosis in 12% of cases. The pathological reinvestigation was ∼93% in favor of the machine learning prediction, demonstrating the power of this approach for correct diagnosis. This methylation profiling classification tool, while for research use and not yet clinically validated, is aimed at generating molecular classification results for treating physicians. The authors developed an interactive website (https://www.molecularneuropathology.org/mnp) that allows researchers to upload their own Illumina Human Methylation BeadChip results and have the sample(s) classified against the references and DNA methylation classification, *MGMT* methylation status, and copy number variation (CNV) returned. Since release, this neuropathology classifier web service has already classified more than 16,000 samples (source from website). To register the classifier as an IVD would be arduous, but clearly this approach has clinical utility and is being adopted by the neuro-oncology community.

### Liver Cancer

The HCCBloodTest developed by Epigenomics is a diagnostic blood test for the detection of hepatocellular carcinoma in cirrhotic patients. This duplex real-time PCR based CE-IVD qualitatively detects methylated *SEPT9* DNA, where hypermethylation is indicative of liver carcinogenesis. The gene β-actin is measured in parallel and used as an internal control to determine whether there was sufficient DNA input. The sensitivity of this assay to detect hepatocellular carcinoma is 91% with 87% specificity, based on an initial and replication study (Oussalah et al., 2018) which collectively had 289 patients with cirrhosis and 98 of them having HCC. The test now forms the basis of an ongoing clinical trial on an estimated 220 patients with either clinically-diagnosed cirrhosis without HCC (confirmed by medical imaging) or cirrhosis patients with early-stage HCC.

### Lung Cancer

Epi proLung^®^, a CE-IVD DNA methylation test developed by Epigenomics for the detection of lung cancer, has been tested in a validation study of 360 clinical specimens from the US and Europe (Weiss et al., 2017). Of these specimens, 152 patients were diagnosed with lung cancer (pathologically confirmed), while the remainder were not diagnosed with lung cancer either after a CT scan or radiological examination and follow-up of the pulmonary nodule. The Epi proLung^®^ IVD is a triplex PCR assay that detects methylated *PTGER4* and *SHOX2*, while β-actin is measured as an internal control for sufficient DNA input (Weiss et al., 2017). The procedure to use the Epi proLung kit is the same as the HCCBloodTest by Epigenomics (see Liver cancer). To classify the presence of lung cancer requires the calculation of an Epi proLung test score (EPLT-Score) which aggregates real-time PCR cycle threshold (Ct) values for triplicate assays of *SHOX2* and *PTGER4* into a compound formula. Different EPLT score thresholds result in different performance characteristics, where an EPLT score of −0.43 has a sensitivity of 59% and specificity of 95%, while an EPLT score of −1.85 has a sensitivity of 85% and specificity of 50%.

### Prostate Cancer

Population screening using blood levels of PSA has long been used for the early detection and treatment prostate cancer. Although originally used as a marker for recurrent prostate cancer, PSA was eventually adopted by the medical community as a standalone screening test. PSA has a reported specificity of 91% and sensitivity of 21% for primary diagnosis of prostate cancer (with cut-off value of 4 ng/ml) (Brawer et al., 1992; Catalona, 1993). While the use of PSA for screening has led to a decrease in mortality rates, this has come at the expense of tremendous over-diagnosis and subsequent over-treatment of the at-risk population. New data shows that prostate cancer treatment may be unnecessary in anywhere from 2% to 67% of cases with PSA detecting a large number of tumors that are unlikely ever to impact the patient. Given the risk of invasive procedures and serious impact on quality of life reported by patients following prostate cancer treatment (radical prostatectomy) there is an urgent need for better biomarkers in the prostate cancer space. Active surveillance of at-risk patients with repeat PSA measures (quarterly), annual examinations by a physician and regular (3 yearly) scans and biopsies has become the method for treating men with an evidently low-grade tumor. Active surveillance minimizes the risk of over-treatment but depends on the rapid detection of changes in tumor grade or growth. At early stages, the probability of a biopsy collecting a tumor sample may be low as any cancer will be just a small percentage of the total prostate mass. This could lead to missed tumor development at biopsy resulting in delayed time to treatment and potentially decreasing rates of survival. As such, the only widely available epigenetic test in prostate cancer has positioned itself to improve cancer detection at biopsy.

The ConfirmMDx test offered as an LDT by MDxHealth targets regions of DNA associated with the genes *GSTP1*, *RASSF1*, and *APC* that exhibit increased methylation in cancer. However, ConfirmMDx does not require a cancer positive biopsy. The target genes all have reported field effects, that is DNA methylation is altered in normal tissue adjacent to the tumor site. By making use of this biology, ConfirmMDx can be used to verify that a tumor negative biopsy is associated with negative risk. MDxHealth report that this results in greater confidence and reduced need for frequent biopsy. ConfirmMDx has an NPV of >90% for high-grade cancers, in Caucasian and African American cohorts (Van Neste et al., 2016; Waterhouse et al., 2019)

### Multiple Cancers

IvyGene (Laboratory for Advanced Medicine) is a test that quantifies the presence of four ubiquitous cancers (breast, colon, liver and lung) by assaying the methylation status of cfDNA from patients’ blood samples across a panel of 46 markers (Hao et al., 2017). This test is marketed as an adjunct clinical test, ordered by physicians to bolster patient observations and available in the USA as an LDT.

Cancer of unknown primary (CUP) origin is a highly heterogenous cancer classification and a particularly frustrating diagnosis for oncologists (Fizazi et al., 2015). Historically, CUP has accounted for anywhere from 3%-9% of all cancer diagnoses (Pavlidis and Pentheroudakis, 2012; Varadhachary and Raber, 2014; Fizazi et al., 2015). However, recent years have seen an apparent decrease in CUP rates to <2% (Urban et al., 2013; Rassy et al., 2019). Despite the rapidly decreasing diagnosis, CUP remains difficult to treat with often poor prognostic outcome (Urban et al., 2013; Fizazi et al., 2015). This is largely due to the fact that CUP is diagnosed only after metastasis and without knowledge of the underlying primary tissue biology. The EPICUP™ assay (Moran et al., 2016) as offered by Ferrer has been developed with the intent of offering these patients more specific diagnoses. EPICUP™ received CE marking in 2015 can be performed using either fresh frozen or FFPE biopsy tissue which is assayed using the Illumina HumanMethylation450 BeadChip. The methylome signature from the BeadChip is used to predict the original tissue of origin and other biological features and to facilitate better treatment decisions. In a multicenter retrospective analysis, this tumor type classifier could predict primary cancer of origin in 87% of patients with a CUP diagnosis (Moran et al., 2016). It should be noted that the underlying platform of this assay (HumanMethylation450 BeadChip) has since been superseded by the more comprehensive Infinium MethylationEPIC BeadChip (Pidsley et al., 2016) and that at the time of writing, Ferrer does not seem to offer an updated product. Still, this assay underlines the unique power of methylome analysis to classify tumors.

GRAIL is a company to watch in the multiple cancer DNA methylation-based IVD space. While only formed in January 2016, they are very well resourced, and their research and clinical program is expansive. At the 2018 American Society of Clinical Oncology (ASCO) annual meeting, GRAIL presented data from their Circulating Cell-free Genome Atlas (CCGA) study showing that WGBS outperformed whole-genome sequencing (WGS) in identifying cancer in a large population of 1627 prospectively collected blood cfDNA samples (Klein et al., 2018). The data were from 749 controls and 878 participants with newly diagnosed untreated cancer across 20 tumor types and all stages. For eight tumor types, the reported sensitivity across stage I-III cancers was 66% colorectal (n = 28), 63% esophageal (n = 19), 56% head and neck (n = 5), 80% hepatobiliary (n = 5), 59% lung (n = 73), 77% lymphoma (n = 17), 73% multiple myeloma (n = 11), 90% ovarian (n = 10), and 80% for pancreatic (n = 10) tumors. In each instance, specificity was held at 95%.

## Translation

The path to clinical translation is long and expensive. The steps involved after development typically include initial testing in cohorts, then clinical evaluation in clinical trials, followed by manufacture of the test and development of processes for its use and finally review by regulatory authorities. The proposed IVD must offer a multitude of benefits over current practice to attract the significant investment required to translate. In addition to product-market fit, the strength of intellectual property, the robustness, and quality of the clinical evidence to present to payers and the nature of the regulatory landscape in the proposed marketplace are all crucial factors in attracting investment. Medical professionals must also be willing to adopt the test, so the utility and clinical evidence needs to be published in peer reviewed scientific and medical publications and presented at conferences and seminars. This section discusses the establishment of strong IP, and how to produce high quality clinical evidence for regulators, payers, and medical professionals.

### Intellectual Property

Patenting in the epigenetics space has sharply risen since around 2000, driven mostly by the patenting of novel diagnostics and epigenetic techniques (Noonan et al., 2013). Patenting provides 20-year exclusivity for companies to exploit ownership of biomarkers and represents a key strategy for biotech and pharma companies to recover costs associated with developing IVDs for clinical utility, for example through licensing fees that would enable labs to implement their test. While important for commercial translation, patent protection can also discourage innovation as it prevents the clinical research community from improving processes to make testing of a biomarker more efficient, for example, quicker testing times, improvements in sensitivity or test accuracy. In some instances, patent protection can generate a monopoly on testing services encouraging excessively high prices out of reach of the general population, e.g. BRCA testing by Myriad. The social and economic implications of biomarker patenting have long been the subject of philosophical debate (Sawyers, 2008; Hopkins and Hogarth, 2012). The patenting of diagnostics has now become far more difficult in the USA after the Association for Molecular Pathology v. Myriad Genetics, Inc court decision in 2013 and the Mayo Collaborative Services v. Prometheus Labs, Inc court decision in 2012 (Dreyfuss et al., 2018). The ruling finds that diagnostics methods based upon biological correlations are not novel but “laws of nature.”

In reaction to the court rulings, the biopharma industry has sought new strategies to describe the uniqueness and inventiveness of their intellectual property. If it is easier to demonstrate novelty and human reasoning in new detection method development, then USA-based diagnostics companies can be expected to formulate strategies coupling promising new biomarkers with a novel (and patentable) detection method. Companies outside of the USA may seek to establish IP in the European and/or Asia-Pacific markets where these court decisions do not apply. For smaller companies seeking capital, uncertainty over the validity of Patent Cooperation Treaty (PCT) claims may make it harder to fundraise in the USA until the key patent claims have been interpreted by the United States Patent and Trademark Office once the PCT reaches national phase. Certainty around the worth of an IP portfolio would come only after commentary is received from the patent examiner and this can be a number of years after initial filing. Some legislators are now seeking to reduce the influence of the earlier court rulings and favor the patentability of biomarkers with the announcement in May 2019 of a bipartisan, bicameral draft bill intending to reform Section 101 of the Patent Act (US Senate, 2019).

### Regulation

While groups such as the International Medical Device Regulators Forum (IMDRF; http://www.imdrf.org) with 10 members jurisdictions are working toward global harmonization of regulation around IVDs, in the foreseeable future large regulatory differences will remain, adding significantly to the complexity of translating a diagnostic assay. The prioritization of geographies is important to consider early in the translation process as the appropriate dossiers of evidence need to be tailored for the regulator. An in-depth analysis of the regulatory systems is beyond the scope of this review, but a brief summary and considerations relating to the USA and European markets will follow. Resources are available elsewhere which consider regulation from an international perspective (Theisz, 2015).

In the United States three different regulatory paths exist for obtaining FDA approval of an IVD. The 510(k) regulatory path is for new tests substantially equivalent to an existing predicate test, while tests with no predicate on the market are subject to *de novo* classification for lower risk tests, or premarket approval (PMA) if they are high risk, such as cancer diagnostics. If the test is done “in-house” in a designated laboratory for patient samples ordered by a physician, then the test can be potentially marketed under “home brew” guidelines, known within the USA as LDTs. Clinical laboratories which run LDTs are regulated by CMS through the Clinical Laboratory Improvement Amendments of 1988 (CLIA) Act. CMS can also approve other methods of certification such as from the state licensing schemes or other organizations such as the College of American Pathologists (CAP). The CLIA regulation concerns the standards of the laboratory and the analytical validity (accuracy and precision) of the test *via* a biennial survey and a laboratory may start distributing test results before evaluation. The CMS’ CLIA program does not address the clinical validity of any test; which is the accuracy of the test to identify, measure, or predict the presence or absence of a clinical condition or predisposition in a patient. The FDA has signaled it intends to increasingly regulate LDTs due to their increasing complexity (Food and Drug Administration, 2014). The FDA guidance shows an intention to introduce to LDT regulation earlier, more robust verification of analytical validity and a requirement for clinical validity. The FDA is also introducing the concept of high-, medium-, and low-risk LDTs and does not intend to regulate low-risk LDTs, nor tests for unmet needs or rare diseases. Cancer diagnostic tests will be classified as high-risk LDTs, so diagnostics under development now should prepare to demonstrate both analytical and clinical validity, regardless of the choice of the PMA or LDT pathway.

In Europe, the In Vitro Medical Devices Directive (IVDD) 98/79/EC was established in 1998 to harmonize standards of conformity and assessment procedures and to help create a unified pan-European market for IVDs. CE Marking is required for all IVDs sold in Europe. CE Marking indicates that an IVD device complies with the IVDD. Under this legislation, an IVD manufacturer only has to self-declare that the product complies with the essential requirements of relevant European laws. With continued evolution in the IVD marketplace, the European Commission recognized amendments were necessary. Starting from public consultations from 2008 onwards, the new In Vitro Diagnostic Medical Devices Regulation (IVDR) (EU) 2017/746 emerged and the legislation entered into force on 26 May 2017, with a 5-year transition period to full implementation on 26 May 2022 (European Parliament, 2017). There is no grandfathering on presently regulated IVDs, so all existing regulated IVDs need to be CE Marked again.

The IVDR has more alignment with International Organization for Standardization (ISO) guidelines and introduces a risk-based classification system with increased oversight by Notified Bodies. The classes are based on the Global Harmonization Task Force classification scheme (predecessor to the IMDRF) and identifies four risk classes A-D, with Class D the highest risk. IVDs for screening, diagnostics, and staging of cancer are classified as Class C and require a full quality management system. “In-house” tests made and used within a single health institution do not have to comply with the IVDR but they require laboratory compliance with EN ISO 15189 (Medical laboratories, Requirements for quality and competence) and the health institution must justify the use of such a test by demonstrating that no commercially available alternative exists. The IVDR also requires compliance with General Data Protection Regulation (GDPR) for use of samples for regulatory purposes (European Union, 2018). Compliance with this regulation also needs to be considered early in the planning of clinical trials. Compared to the IVDD, the IVDR also has stronger analytical performance requirements for diagnostic tests, including the requirement for reference materials and methods.

### Quality Management Systems

Any IVD seeking registration needs to provision a Quality Management System (QMS) and comply with Good Manufacturing Practice (GMP) requirements. This provides the framework for conformity assessment and ongoing post-market responsibilities such as quality control, external quality assurance, and adverse event reporting. Each jurisdiction has different conformity assessment procedures. With global harmonization in mind, the IMDRF began the Medical Device Single Audit Program (MDSAP) initiative in 2012. Regulatory authorities within the working group have implemented a program where auditing organizations can conduct a single audit of a medical device manufacturer that would be accepted by multiple regulators to address QMS and GMP requirements.

For PMA submissions in the USA, the FDA needs to be satisfied that the appropriate design and manufacturing controls are present and has the power to undertake a pre-approval inspection and will schedule a post-approval inspection with 8–12 months of approval. LDTs do not have to comply with FDA quality system regulation, nor be subject to FDA inspection. LDTs (also known as “in-house” IVDs in other jurisdictions) are regulated around the compliance of the laboratory network. Many countries have made ISO 15189 part of their mandatory medical laboratory accreditation requirements, however in the USA, accreditation to the ISO 15189 standard does not meet CLIA requirements and cannot replace a CLIA-based accreditation (Schneider et al., 2017). Similar to the regulation pathway, priority of jurisdictions for translation should inform the design of the QMS. The existing standards also change over time and new standards are introduced. Relevant to cancer diagnostics, a new ISO standard on the “Requirements for evaluating the performance of quantification methods for nucleic acid target sequences — qPCR and dPCR” (ISO/FDIS 20395) is now under development (International Organization for Standardization, 2019).

When planning translation of a diagnostic test, it is critical to understand what is the appropriate design and evidence for regulatory bodies that adequately supports analytical and clinical validity. The regulators must also be satisfied that this evidence is gathered from the intended target population of the IVD. For guidance on constructing the appropriate dossier of evidence, the Clinical and Laboratory Standards Institute (CLSI), a non-profit organization, produces a set of guidelines relevant to the diagnostics industry. Their “Evaluation of Detection Capability for Clinical Laboratory Measurement Procedures” guideline document is intended for use by IVD manufacturers, regulators, and clinical laboratories to provide guidance for the evaluation and documentation of the detection limits of clinical laboratory measurement procedures (Clinical and Laboratory Standards Institute, 2012).

## Future

Presently, there is a flurry of activity in developing DNA methylation-based IVDs. The attention to this sector will only increase with recent announcements by companies such as GRAIL, who found that methylome sequencing of cfDNA outperformed somatic mutation sequencing for primary diagnosis of cancer. An emerging trend is the incorporation of larger panels of methylated biomarkers for multi-cancer detection and determining the tissue of origin. There are now several studies showing that the methylation state of circulating DNA can be used to predict tissue of origin (Kang et al., 2017; Moss et al., 2018), with spin out companies such as EarlyDiagnostics translating these findings. A large plasma cfDNA panel of 9223 CpG sites designed using The Cancer Genome Atlas (TCGA) data has been shown to detect common advanced cancers and underlying cancer type with high accuracy (Liu et al., 2018). Researchers in partner with AnchorDx Medical (Guangzhou, China) have recently shown that a panel of nine bisulfite sequencing amplicons can detect in plasma early stage lung cancer with high sensitivity (Liang et al., 2019).

Another emerging trend in the cancer IVD sector is the development of multi-analyte tests, such as the CancerSEEK test, which combines somatic mutation detection and immunoassays (Cohen et al., 2018). Recently, Guardant Health acquired Bellwether Bio which will allow them to include nucleosome positioning and fragmentomics information with their NGS ctDNA analysis (Snyder et al., 2016). This study of cfDNA fragment length, an indirect measure of nucleosome positioning, has recently been shown to have good clinical utility (Cristiano et al., 2019).

With the continued reduction in the cost of NGS, the use of whole methylomes for biomarker discovery is becoming more commonplace. With sufficient subjects and sequencing depth, all high utility biomarkers will be identified in a screen. Some tests under development, such as the UroMark 150 biomarker assay for bladder cancer detection, are basing the IVD readout on NGS.

There are many new innovations in determining the methylation state of DNA. Two new enzyme-based DNA conversion methods, Enzymatic Methyl-seq (New England Biolabs, 2019) and TET-assisted pyridine borane sequencing (TAPS) (Liu et al., 2019) make use of enzymes to convert the DNA and with these gentler conditions, may offer more recovery of amplifiable DNA than bisulfite-treatment and the resultant DNA is suitable as input for targeted as well as NGS-based approaches. The continued development of third generation sequencing technology such as that from Pacific Biosciences or Oxford Nanopore Technologies offers new opportunities for direct epigenetic detection. To this end, three groups have trained and tested machine-learning approaches to detect methylated DNA on Oxford Nanopore Technologies MinION devices with reasonable classification success (Rand et al., 2017; Simpson et al., 2017; Ni et al., 2019). However, low-input, short cfDNA fragments are not an optimal fit for these long-read platforms. Methylscape, a new method to directly detect and partition methylated DNA using physicochemical properties is also an exciting innovation and offers the potential for an inexpensive pan-cancer test (Sina et al., 2018).

The continued technological development and increasing commercialization activity in the DNA-methylation IVD sector are leading to a fast-paced, innovative, and competitive environment that will result in significant benefits to patients for the early detection and management of cancer.

## Author Contributions

JR conceptualized the review and led the writing process. JR, WL, DG, CM, YL, KD, and KF performed the literature search and wrote the manuscript. All the authors have read and approved the manuscript.

## Funding

This work is completely funded by the Commonwealth Scientific and Industrial Research Organisation (CSIRO), the Australian national science agency. CSIRO Health and Biosecurity receives payment from Clinical Genomics and Rhythm Biosciences for translated IVDs. Clinical Genomics and Rhythm Biosciences had no role in review design, data collection and analysis, decision to publish, or preparation of the manuscript.

## Conflict of Interest

The authors declare that the research was conducted in the absence of any commercial or financial relationships that could be construed as a potential conflict of interest.
